# Intraductal Tubulopapillary Neoplasm Diagnosed Before Surgery: A Case Report

**DOI:** 10.7759/cureus.36932

**Published:** 2023-03-30

**Authors:** Yoko Senaha, Sadamu Takahashi, Kazushi Hara, Kotaro Yoshida, Makoto Nagasaki

**Affiliations:** 1 General Medicine, National Hospital Organization Hamada Medical Center, Hamada, JPN; 2 Surgery, National Hospital Organization Hamada Medical Center, Hamada, JPN; 3 Radiology, National Hospital Organization Hamada Medical Center, Hamada, JPN; 4 Pathology, National Hospital Organization Hamada Medical Center, Hamada, JPN

**Keywords:** immunohistochemical, endoscopic ultrasonography fine needle aspiration, neoplasm, pancreas, intraductal tubulopapillary neoplasm

## Abstract

Intraductal tubulopapillary neoplasm (ITPN) is a rare disease in the pancreas with a better prognosis than pancreatic ductal adenocarcinoma (PDAC) and a different treatment strategy. Therefore, it is important to confirm its diagnosis before the surgery. However, few cases have been diagnosed preoperatively. In this report, we present a case of ITPN that was successfully diagnosed preoperatively.

A 70-year-old female patient was incidentally diagnosed with a pancreatic tumor. The patient was asymptomatic, and her blood tests were all within the normal range. A dynamic computed tomography scan showed an indistinct mass with small cysts and a dilated pancreatic duct. The mass was well contrasted in the arterial phase. These findings were not enough to confirm ITPN. Therefore, endoscopic ultrasonography fine needle aspiration biopsy was performed. The specimen had no mucin and the neoplastic cells exhibited a tubulopapillary growth pattern. Moreover, the neoplastic cells were immunohistochemically positive for MUC1, CK7, and CK20, but negative for MUC2, MUC5AC, synaptophysin, and Bcl-10. Consequently, the preoperative diagnosis was confirmed as ITPN. Hence, a subtotal-stomach-preserving pancreaticoduodenectomy was performed, and the patient had a good postoperative course and was discharged after 26 days. Tegafur, gimeracil, and oteracil were administered as postoperative adjuvant chemotherapies for one year. Seventeen months after the surgery, no recurrence has been detected. ITPN and PDAC have different prognoses and treatment strategies. In this report, we experienced a case of ITPN preoperatively diagnosed and successfully treated.

## Introduction

Intraductal tubulopapillary neoplasm (ITPN) is newly classified by WHO as an intraductal tumor of the pancreas [1]. Yamaguchi et al. reported that ITPN accounted for only 0.9% of the total exocrine pancreatic tumors and 3% of the total pancreatic intraductal tumors [2]. Recently, ITPN has been gradually recognized and the number of reports is increasing. However, few cases have been diagnosed preoperatively. In some cases, surgery was performed with a suspicion of either pancreatic ductal adenocarcinoma (PDAC) or intraductal papillary mucinous neoplasm (IPMN) [3-5]. Some articles previously reported that the prognosis of ITPN is better than the conventional PDAC [6-8]. In Japan, neoadjuvant chemotherapy (NAC) is commonly performed for PDAC [9]. However, its effectiveness for ITPN has not been demonstrated, and surgical treatment is usually applied [10]. Therefore, the concept of recognizing and diagnosing ITPN before surgery is important. In this report, we present a case of ITPN that was successfully diagnosed before surgery.

## Case presentation

**Figure 1:**
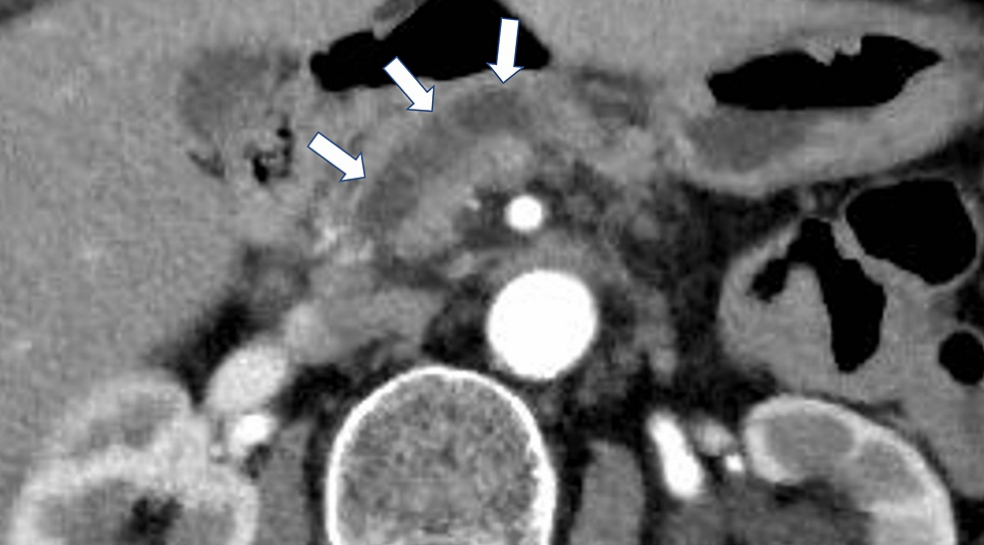
Dynamic CT showed dilatation of the main pancreatic duct (white arrows).

**Figure 2:**
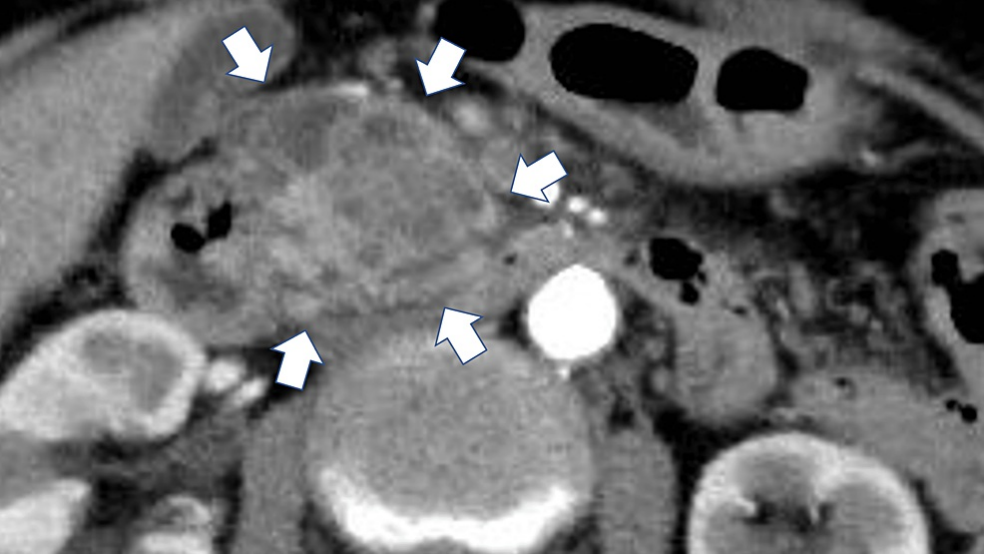
Dynamic CT showing a mass (d = 30mm) with few small cysts in the head of the pancreas (white arrows).

A 70-year-old female visited our hospital for a periodic routine checkup with a medical history of appendicitis, ovarian cyst, hypertension, and dyslipidemia. Her anthropometric measures were as follows; height, 1.52 m; weight, 42.6 kg; and body mass index (BMI), 18.4 kg/m^2^. The patient did not consume alcohol but smoked one pack of cigarettes daily for 50 years. Furthermore, she was asymptomatic, and her blood tests such as CEA, CA19-9, Span-1, and DUPAN-2 were all within the normal range. Dynamic computed tomography (CT) scan showed the main pancreatic duct (MPD) dilatation (Figure 1) and an indistinct mass with a diameter of 30 mm in the pancreatic head. Small cysts inside the mass were detected (Figure 2). The CT values of tumor lesions were 35.9 Hounsfield Unit (HU) in the unenhanced phase, 60.7 HU in the arterial phase, and 70.4 HU in the venous phase.

**Figure 3:**
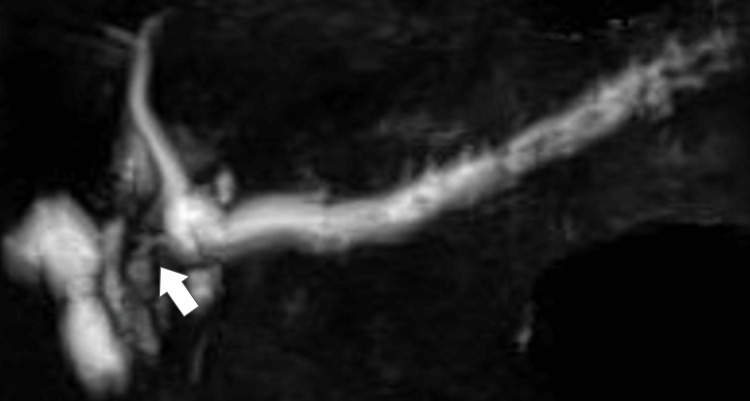
MRCP showing MPD interrupted in the head of pancreas (white arrowhead) and dilating in the peripheral side.

**Figure 4:**
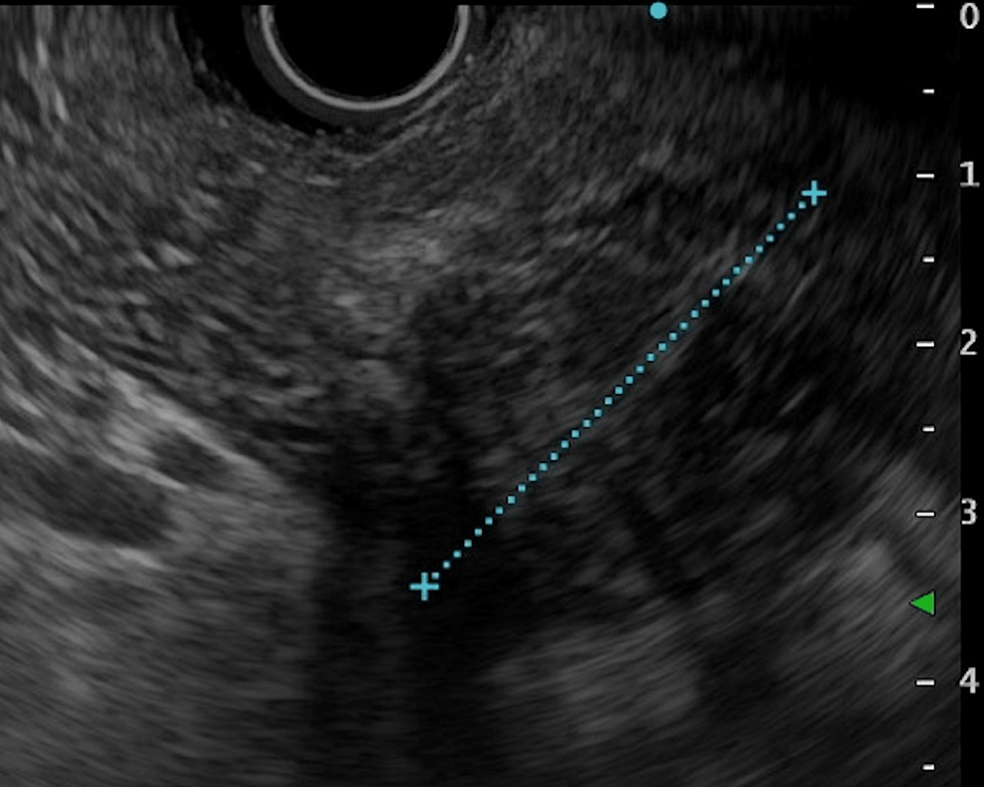
EUS showing a solid mass.

**Figure 5:**
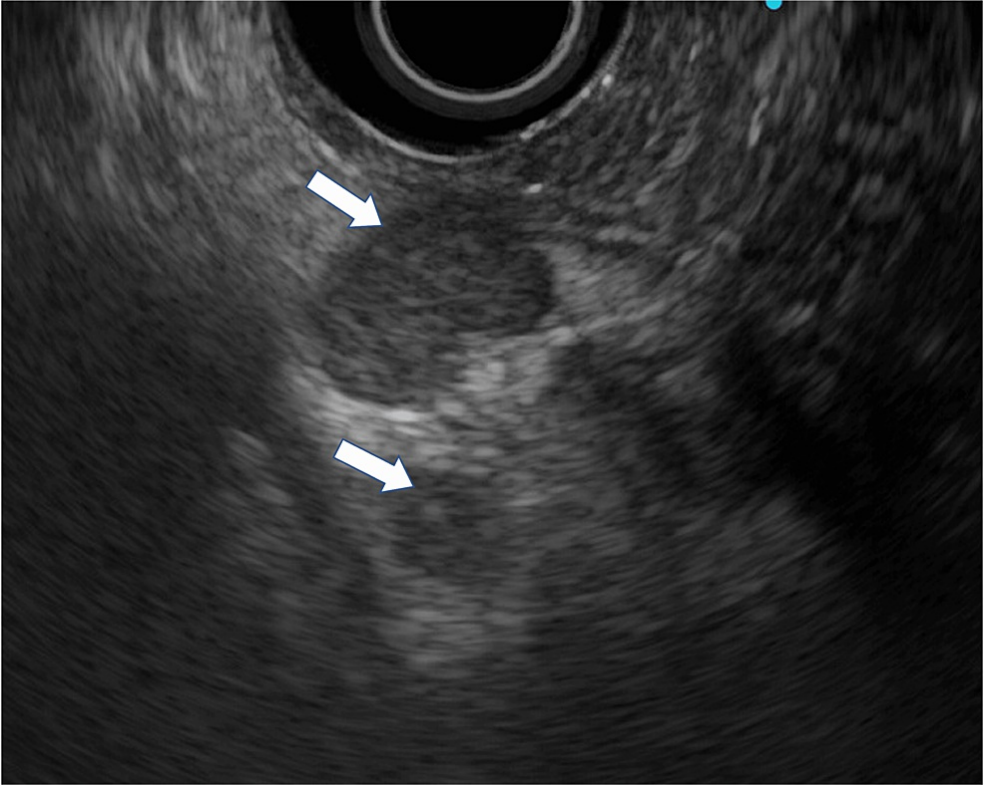
EUS showing partial cysts in the mass (white arrowheads).

The magnetic resonance cholangiopancreatography (MRCP) revealed that MPD was interrupted in the head of the pancreas and dilated more peripherally (Figure 3). The CT scan indicated a lack of evidence of distant metastasis and swollen lymph nodes. Thus, an endoscopic ultrasonography fine needle aspiration (EUS-FNA) biopsy was applied to make a preoperative diagnosis. Endoscopic examination showed no mucin secretion in the vater papilla. EUS, similar to CT and magnetic resonance imaging (MRI), showed a solid and partially cystic mass in the pancreatic head, and the peripheral side of the pancreatic duct was markedly dilated (Figures 4, 5).

**Figure 6:**
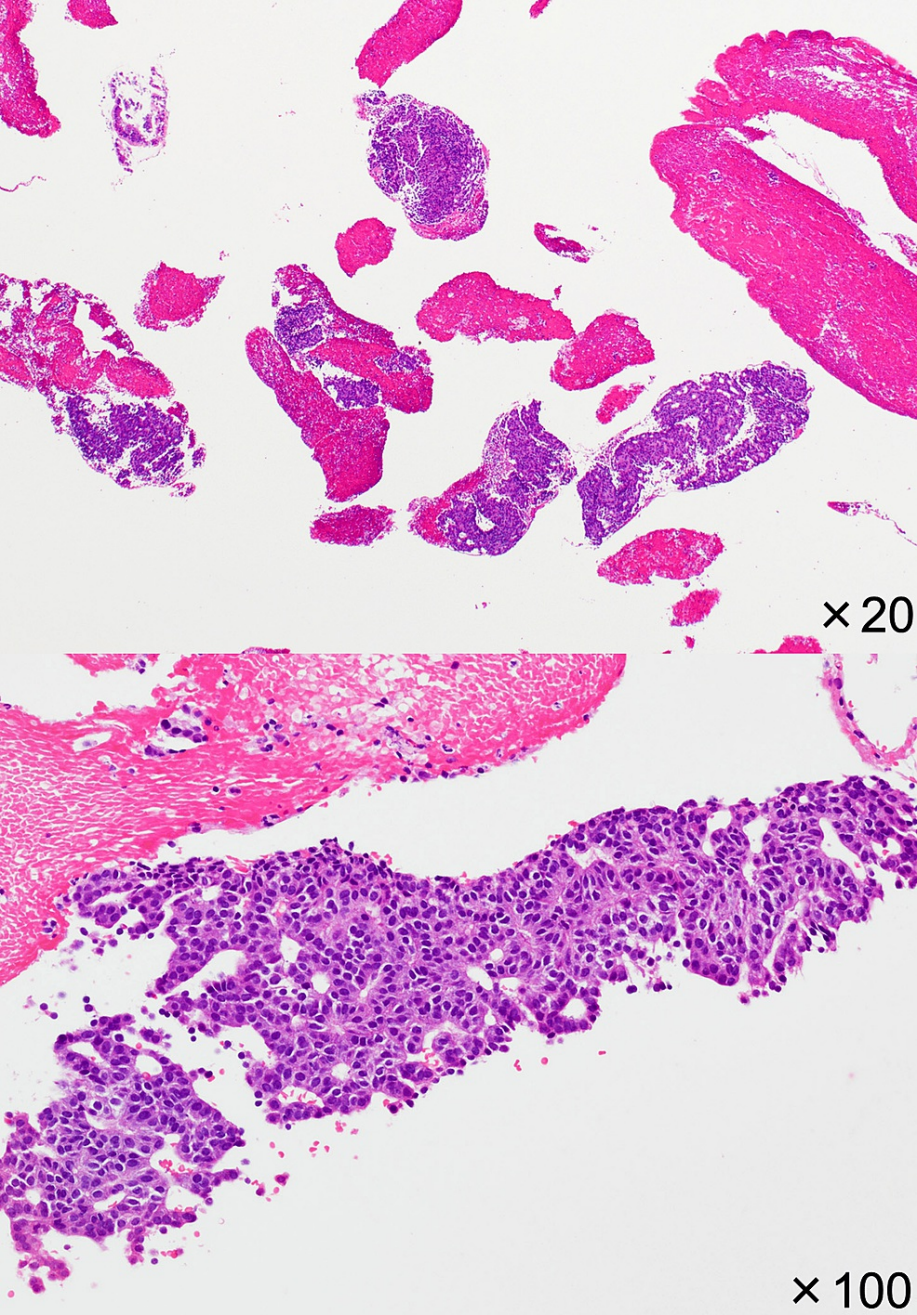
Neoplastic cells exhibiting tubulopappillary growth patterns; microscopic findings (H&E, 20× and 100×).

**Figure 7:**
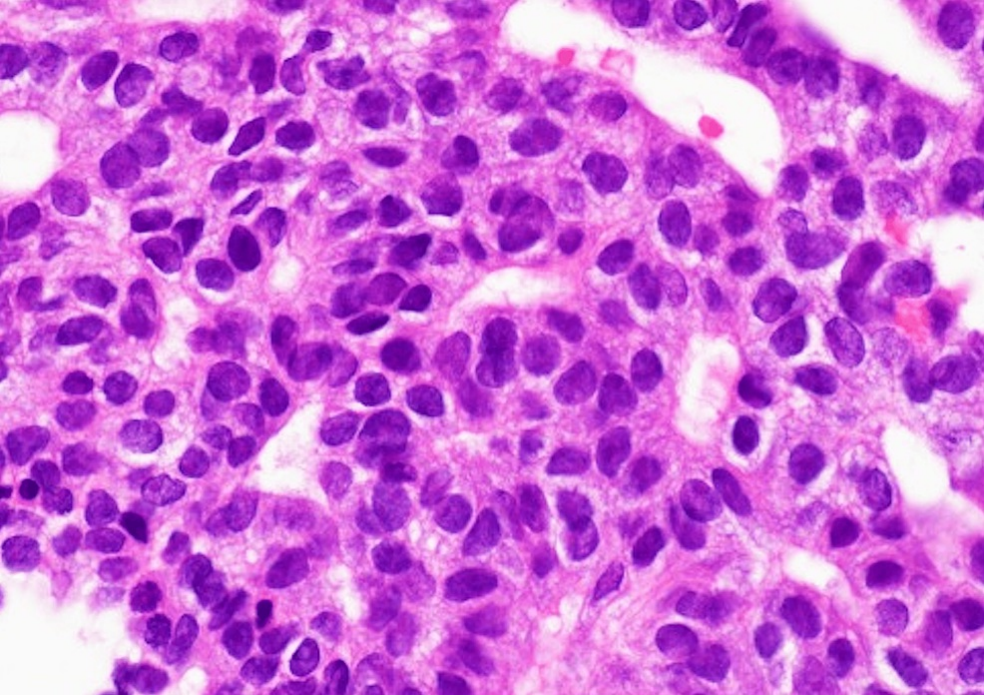
Neoplastic cells showing mild nuclear atypia The cells contain redoubled nuclear size and elevated N/C ratios; microscopic findings (H&E, 400×).

**Figure 8:**
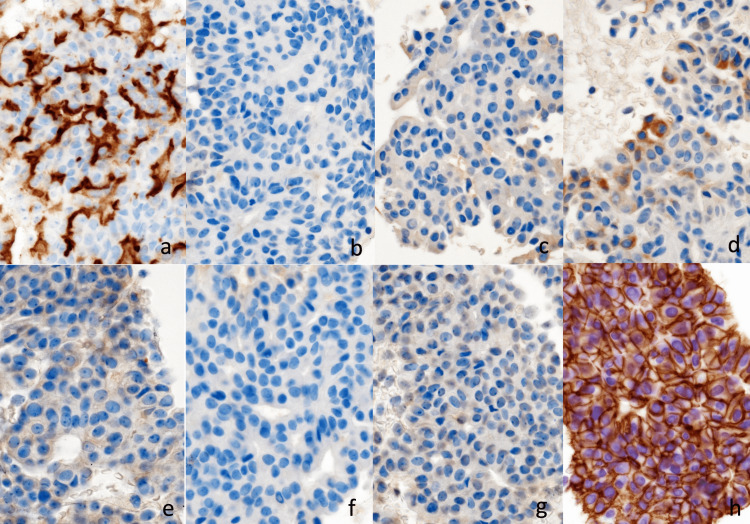
Immunological findings of EUS-FNA (×400). Immunohistochemically positive for MUC1, and the membranous staining of β-catenin, and negative for MUC2, MUC5AC, MUC6, synaptophysin, chromogranin A, and Bcl-10. (a) MUC1, (b) MUC2, (c) MUC-5AC, (d) MUC6, (e) synaptophysin, (f) chromogranin A, (g) Bcl-10, (h) β-catenin (membrane positive)

At the microscopic findings of the specimen, neoplastic cells exhibited a tubulopapillary growth pattern in the pancreatic duct (Figure 6) without mucin secretion. The nuclear atypia was mild (Figure 7). These findings implied the possibility of ITPN, neuroendocrine carcinoma (NEC), acinar cell carcinoma (ACC), solid pseudopapillary neoplasm (SPN), and metastatic cancer. Nevertheless, neoplastic cells were found to be immunohistochemically positive for MUC1, CK7, CK20, and the membranous staining of β-catenin, and negative for MUC2, MUC5AC, MUC6, synaptophysin, chromogranin A, CD56, CD10, CEA, TTF-1, PAX8, and Bcl-10. Additionally, p53 expressed 1%, and the Ki-67 labeling index was 17%. Consequently, the preoperative diagnosis was ITPN (Figures 8a-8h).

**Figure 9:**
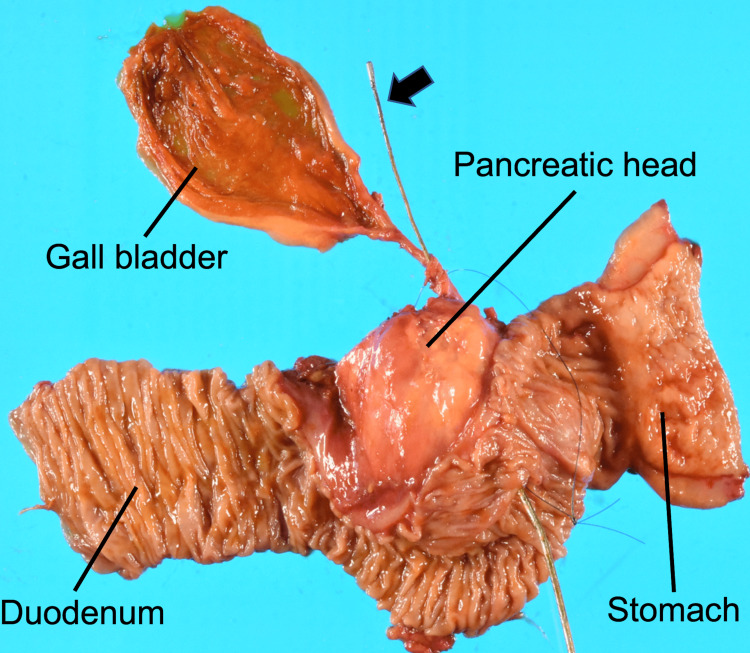
Resected specimen of a hard nodule touching the pancreatic head. Sonde through the pancreatic duct (black arrow).

**Figure 10:**
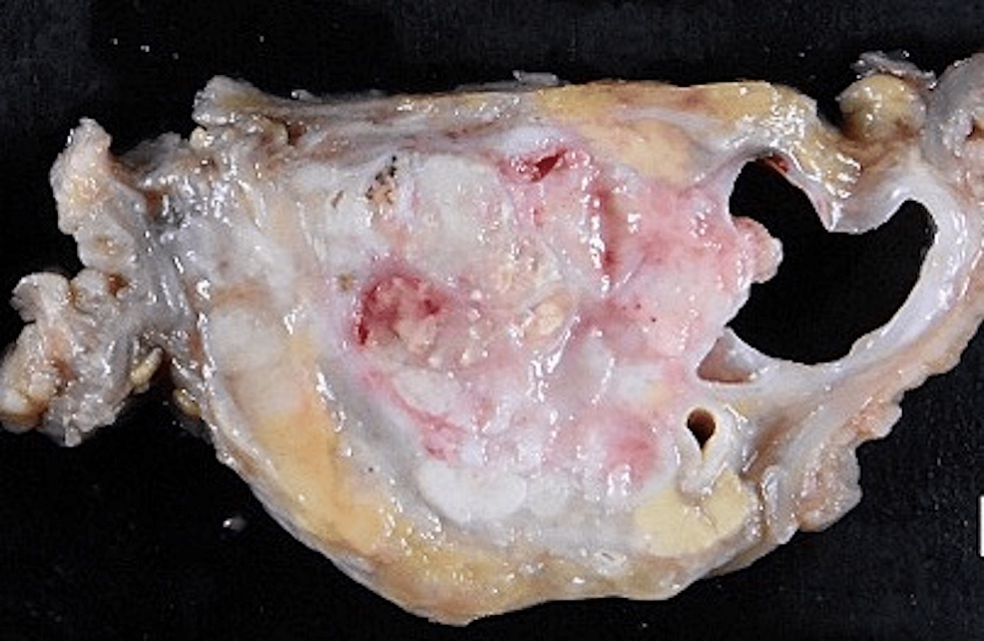
At macroscopic findings, it was a solid tumor and there was no mucin.

Therefore, a subtotal-stomach-preserving pancreaticoduodenectomy (SSPPD) with lymph node dissection was performed. A hard nodule was touched in the pancreatic head of the resected specimen (Figure 9). Macroscopic findings of the specimen showed a solid tumor of 4.9 cm in diameter localized in the pancreatic head with a lack of mucin (Figure 10).

**Figure 11:**
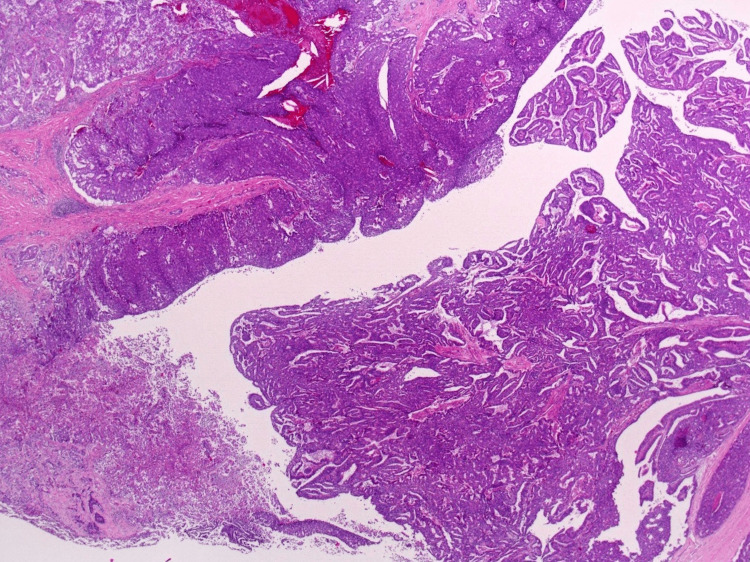
Neoplastic cells exhibit tubulopapillary growth pattern (microscopic findings).

**Figure 12:**
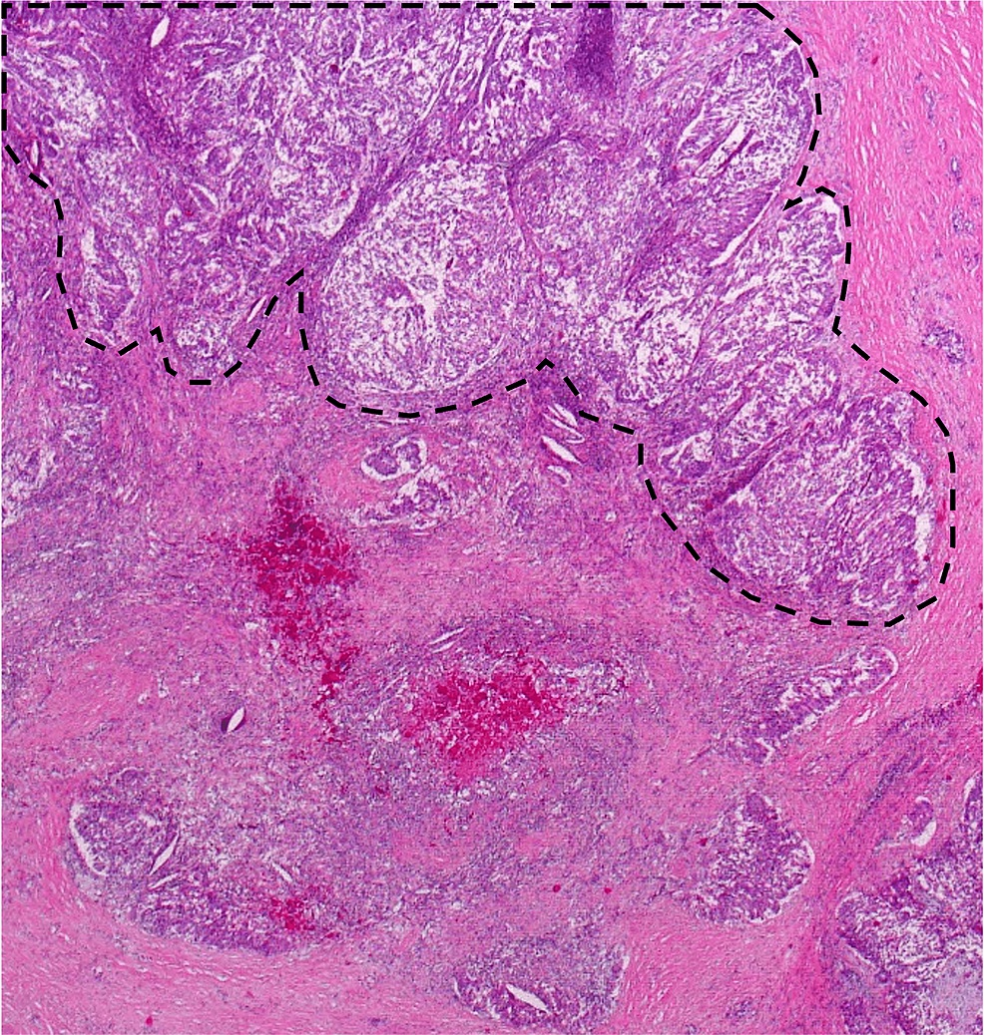
The growth pattern of the invasive component. This is an expanding pattern characterized by a distinct border with the surrounding tissue (marked with black dashed lines).

**Figure 13:**
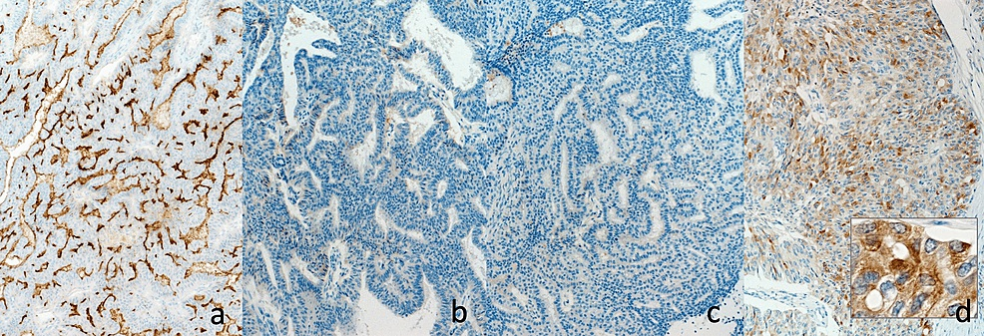
Immunohistochemically findings of resected specimen (×100). Immunohistochemically positive for MUC1 and MUC6, and negative for MUC2 and MUC-5AC. a. MUC-1, b. MUC-2, c. MUC-5AC, d. MUC-6 (inserted, ×400)

At microscopic findings, neoplastic cells exhibited a tubulopapillary growth pattern in the pancreatic duct and included some invasive components (Figure 11). The most dominant growth pattern at the neoplasm margin is an expanding one characterized by a distinct border with the surrounding tissue (Figure 12). Some necrotic tissues were also observed. Immunohistochemically findings were similar to the results of the EUS-FNA biopsy (Figures 13a-13d). The definitive diagnosis was invasive ITPN (pT2N0M0).

No postoperative complications were reported, and the patient was discharged 26 days after her surgery. Starting day 39 post-surgery, she underwent chemotherapy for one year with tegafur, gimeracil, and oteracil (S-1). There was no detected sign of recurrence until 17 months postoperatively.

## Discussion

When treating ITPN, a specific therapeutic strategy different from other pancreatic tumors, such as PDAC, should be applied. In Japan, the cases of resectable PDAC undergo NAC based on Unno et al.'s report [9]. Nonetheless, the benefits of NAC on ITPN have not been recognized yet. Therefore, the basic treatment for ITPN is resection of the tumor [10]. This difference may be because ITPN has a better prognosis than PDAC [7,8,10]. Date et al. reported that the survival rates in overall one, three, and five years postoperatively for ITPN are 97.3%, 80.7%, and 80.7%, respectively [7]. Conversely, similar survival rates of the resected cases with PDAC lacking regional lymph node metastasis are 81.7%, 48.4%, and 33.8%, and those having 1-3 regional lymph nodes metastasis are 72.9%, 26.7%, and 15.2% [6]. Therefore, it is important to differentiate ITPN from PDAC and to confirm the diagnosis preoperatively with biopsy, whenever possible.

Methodology

We conducted an advanced search in Pubmed browser using the words (“Intraductal tubulopapillary neoplasm” OR “ITPN”) AND “pancreas,” between 2009 and October 2022. Consequently, we analyzed the findings of the characteristic images and the preoperative diagnosis.

Motosugi et al. referred to the ITPN characteristic imaging findings as a “two-tone-duct sign,” because, whether in CT or MRI, the dilated pancreatic duct appears in two colors: intraductal tumor and dilated duct without tumor. Moreover, ITPN was reviewed as a “cork-of-wine-bottle sign” due to the MRI findings of the pancreatic juice surrounding the tumor. The report stated that 70% (7/10 cases) showed a “two-tone-duct sign” in dynamic CT, whereas 38% (3/8 cases) presented a “cork-of-wine bottle sign” in MRI [11].

Based on the literature review, four articles [3,5,12,13] mentioned 24 cases with a “two-tone-duct sign” (Table 1). Twenty-five percent (6/24 cases) showed a “two-tone-duct sign” and 87.5% (21/24) had an invasive component, out of which, 23.8% (5/21) showed the sign. Conversely, only 12.5% (3/24) had a noninvasive tumor component, out of which, 33.3% (1/3) showed the sign. Regarding the “cork-of-wine bottle sign,” Sakamoto et al. noted the following: in the case where the tumor occupies the entire lumen of the pancreatic duct, MRI does not show the sign, it rather presents a ductal interruption. Therefore, the sign is not always a confirmed finding depending on the size and the degree of the lesion progression [14].

**Table 1:** Clinical and demographic summary of four article cases and our case presented with “two-tone-duct sign.” M, male; F, female.

Case	Author	Year	Age	Sex	Size (mm)	Invasive	2-tone-duct sign
1	Kim^10)^	2019	47	M	-	-	-
2	Kim^10)^	2019	53	M	18.5&13.3	-	-
3	Kim^​​​​​​​10)^	2019	65	M	13.3	+	-
4	Kim^​​​​​​​​​​​​​​10)^	2019	71	F	15	+	-
5	Kim^​​​​​​​​​​​​​​10)^	2019	53	M	98	+	-
6	Kim^​​​​​​​​​​​​​​10)^	2019	65	F	23.1	+	-
7	Kim^​​​​​​​​​​​​​​10)^	2019	50	F	12.7	-	+
8	Kim^​​​​​​​​​​​​​​10)^	2019	34	M	-	+	-
9	Zhang^11)^	2019	36	F	40	+	-
10	Zhang^11)^	2019	62	F	32	+	+
11	Shimizu^12)^	2020	73	F	25	+	+
12	Khristenko^3)^	2022	36	M	55	+	3 cases were showed the sign.
13	Khristenko^​​​​​​​3)^	2022	61	M	63	+	
14	Khristenko^​​​​​​​3)^	2022	87	F	87	+	
15	Khristenko^​​​​​​​3)^	2022	63	M	54	+	
16	Khristenko^​​​​​​​3)^	2022	65	M	21	+	
17	Khristenko^​​​​​​​3)^	2022	72	F	23	+	
18	Khristenko^​​​​​​​3)^	2022	74	F	30	+	
19	Khristenko^​​​​​​​3)^	2022	58	F	23	+	
20	Khristenko^​​​​​​​3)^	2022	68	M	28	+	
21	Khristenko^​​​​​​​3)^	2022	52	M	41	+	
22	Khristenko^​​​​​​​3)^	2022	75	F	33	+	
23	Khristenko^​​​​​​​3)^	2022	54	M	31	+	
24	Our case	2023	71	F	49	+	-

Consequently, the “two-tone-duct sign” and the “cork-of-wine bottle sign” cannot be considered useful findings in differentiating ITPN from PDAC. In MRI, some articles reported ITPN as high on T2WI and DWI and low on T1WI [3,4,13], whereas PDAC appears to be the same. The concentration of MRI findings is not useful enough to build a differentiating protocol between ITPN and PDAC.

Khristenko et al. reported 12 cases of ITPN in their studies, demonstrating that CT values in the arterial and venous phases are the differentiation keys of the invasive ITPN and PDAC. The mean CT values of the ITPN lesions in the unenhanced, arterial, and venous phases were 39.2 ± 7.9 HU, 62.3 ± 14.6 HU, and 68.0 ± 15.6 HU, respectively. By contrast, the mean CT values of the PDAC lesions in the arterial and venous phases were 34.4 ± 12.3 HU and 40.1 ± 13.6 HU, respectively [3]. In our case, the CT values were 35.9 HU in the unenhanced phase, 60.7 HU in the arterial phase, and 70.4 HU in the venous phase. Alternatively, if the dilated pancreatic duct was filled with pancreatic juice, the CT values become low (almost 0 HU). In our case, the CT values in the dilated pancreatic duct were slightly enhanced in the unenhanced (7.4 HU) and in the arterial phases (18.6 HU). These findings suggest that the tumor growth is within the pancreatic duct. Therefore, the higher CT values of the tumor and the dilated pancreatic duct in the arterial phase are suspicious findings to favor ITPN over PDAC.

Although the imaging findings can assist with the diagnosis, still, they are insufficient. Microscopic and immunological findings of the EUS-FNA biopsy are necessary for the preoperative diagnosis [15]. In our case, the microscopic findings of the biopsy showed that neoplastic cells exhibited tubulopapillary intraductal growth patterns with mild nuclear atypia without mucin secretion. These findings are characteristics of ITPN rather than PDAC [15,16].

Moreover, our immunological findings were positive for MUC1 and MUC6 and negative for MUC2 and MUC5-AC [15,17]. These characteristics are useful for IPMN differentiation. In case of IPMN, MUC5-AC is always found positive. However, in our case, MUC6 was weakly positive. In this regard, Yamaguchi stated that a negative MUC6 is not a definitive reason to rule out ITPN [18]. Therefore, to confirm the diagnosis, other immunological findings should be referred to. Additionally, the alcian blue stain, which shows the secretion of mucin [17], is useful in differentiating IPMN from ITPN. In this regard, IPMN staining is found positive, whereas ITPN is negative. In our case, alcian blue staining was negative.

We considered the possibility of NEC and ACC as other tumors showing similar intraductal growth patterns. NEC is usually positive for synaptophysin and chromogranin [19], whereas ACC is positive for Bcl-10 [20]. The results in our case were negative for both. We also included the possibility of SPN as a differential diagnosis. However, in the case of SPN, significant staining is detected in the nucleus or the cytoplasm for β-catenin [15]. Our case was a membranous staining. Consequently, we were able to diagnose ITPN preoperatively. Biopsy specimens could differentiate ITPN from other pancreatic tumors. Therefore, EUS-FNA should be considered as much as possible [15,16].

After surgery, the specimen of operation revealed growth patterns of neoplasm infiltrating surrounding tissue (INF) [6]. INF indicates the most dominant growth patterns at the neoplasm margin. On the one hand, ITPN shows an expanding pattern of growth characterized by a distinct border with the surrounding tissue (INFa). On the other hand, PDAC shows a diffusely infiltrating pattern of growth characterized by an indistinct border with the surrounding tissue (INFc) [6]. INFa was shown in our case. Such patterns of invasion cannot be determined with few samples on EUS-FNA biopsy. Therefore, these findings are apparently postoperatively but are useful to exclude PDAC.

Postoperative chemotherapy is still not standardized; however, some medicines, such as gemcitabine, gemcitabine-capecitabine, S-1, and 5-Fluorouracil, leucovorin, and oxaliplatin (FOLFOX) are being used [16]. Nevertheless, their efficacy and significance remain unknown. Therefore, it is recommended to establish an effective treatment by further collecting and analyzing cases.

## Conclusions

ITPN has a better prognosis than PDAC. ITPN and PDAC have different treatment strategies. Preoperative differentiation of from other pancreatic tumors, especially PDAC, is very important. Preoperative diagnosis of ITPN may enable treatment without excess or deficiency.
